# Basic Fibroblast Growth Factor‐Releasing Bioabsorbable Polyglycolic Acid Dura Mater Enhances Neural Progenitor Cell Proliferation and Neuroprotection After Brain Injury

**DOI:** 10.1002/brb3.71008

**Published:** 2025-10-29

**Authors:** Yoshiro Ito, Ayako Oyane, Yuji Matsumaru, Eiichi Ishikawa

**Affiliations:** ^1^ Department of Neurosurgery, Institute of Medicine University of Tsukuba Tsukuba Ibaraki Japan; ^2^ Research Institute of Core Technology for Materials Innovation National Institute of Advanced Industrial Science and Technology (AIST) Tsukuba Ibaraki Japan

**Keywords:** adsorption, artificial dura mater, delivery, neural regeneration, neurological function, oxygen plasma treatment

## Abstract

**Background:**

Traumatic brain injury harms health, causes disability, and burdens health care systems and economies. Although new treatments for brain injury have been developed, their therapeutic efficacy remains insufficient. Herein, we demonstrate the therapeutic efficacy of artificial dura mater with varying basic fibroblast growth factor (bFGF)‐releasing capabilities using a brain injury model.

**Methods:**

Artificial dura mater of lower (FGF‐L) and higher (FGF‐H) bFGF‐releasing capabilities was prepared via oxygen plasma treatment for polyglycolic acid nonwoven fabric followed by bFGF adsorption. Mice received either bFGF‐releasing dura mater (FGF‐L, FGF‐H) or bFGF‐free dura mater (FGF‐C) at the site of the induced brain injury.

**Results:**

Neurological functions significantly improved in the FGF‐L and FGF‐H groups compared with those in the FGF‐C group on Day 14. No significant difference was observed in the brain injury area between the FGF‐C group and either the FGF‐L or FGF‐H group. The number of SRY‐box transcription factor 2‐positive cells in the cortex was significantly larger in the FGF‐L and FGF‐H groups than in the FGF‐C group on Day 7. The terminal transferase dUTP nick‐end labeling‐positive cell ratio was significantly lower in the FGF‐H group than in the FGF‐C group on Day 14. The occludin‐positive and ZO‐1‐positive cell ratios were significantly greater in the FGF‐H group than in the FGF‐C group on Day 14, suggesting improved blood–brain barrier integrity.

**Conclusion:**

The bFGF‐releasing dura mater enhanced neural progenitor cell proliferation, inhibited apoptosis and blood–brain barrier breakdown, and contributed to neurological function recovery in brain‐injured mice.

## Graphical abstract

A basic fibroblast growth factor‐releasing artificial dura mater enhanced neural progenitor cell proliferation, inhibited apoptosis, and preserved blood–brain barrier integrity after brain injury. This biomaterial offers a promising strategy for promoting functional recovery by reducing secondary damage in traumatic brain injury.

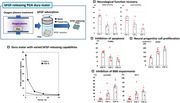

## 1 Introduction

The global average age‐standardized incidence of traumatic brain injury (TBI) was 369 per 100,000 individuals in 2016 (GDB 2016 Traumatic Brain Injury and Spinal Cord Injury Collaborators 2019). TBI harms health, causes disability, and burdens health care systems and economies through productivity loss and high health care costs (Te Ao et al. 2014). TBI consists of primary and secondary injury mechanisms. Primary injury results in localized brain damage, whereas secondary injury is the body's physiological response, including inflammatory reactions caused by blood–brain barrier (BBB) disruption, infiltration of peripheral blood cells, brain edema, and the release of numerous immune‐mediated substances including chemotactic factors and interleukins (Thapa et al. 2021). Conventional treatments aim to prevent secondary injury; however, their efficacy is limited (Carney et al. 2017). New pharmacological approaches, including anti‐excitotoxic, antioxidants, anti‐neuroinflammatory, and antiapoptotic treatments, have yielded poor results (Thapa et al. 2021). Stem cell therapy has emerged as a new therapeutic approach to improve neurological functions via damaged central nervous system regeneration. However, its application faces challenges, including tumorigenicity, contamination, and immune rejection risks, difficulties in large‐scale production and quality control, and high costs (Li et al. 2020; Panos et al. 2024). These limitations highlight the need for more practical and effective therapies.

Some growth factors support neuroprotection and neural regeneration (Burke et al. 2013). Therefore, growth factor‐based therapies may reduce secondary injury and improve TBI outcomes (Atkinson and Dickman 2023). Major challenges with growth factor‐based therapies include a short half‐life (minutes to hours) of growth factors, difficulty of their delivery to the brain, and their low penetration at the target site (Atkinson and Dickman 2023). To address these challenges, a basic fibroblast growth factor (bFGF)‐releasing artificial dura mater was developed by oxygen plasma treatment for a bioabsorbable polyglycolic acid (PGA) nonwoven fabric followed by bFGF adsorption (Ito et al. 2025, 2022). This bFGF‐releasing dura mater can release bFGF for at least 7 days under physiological conditions and is expected to deliver biologically active bFGF directly to the target site of brain while supporting the dural repair. It exerted therapeutic efficacy in focal cerebral infarction in mice (Ito et al. 2025); however, its efficacy for TBI remains uncertain. Therefore, the present study aimed to evaluate the therapeutic efficacy of bFGF‐releasing dura mater using a brain injury mouse model.

## 2 Materials and Methods

### 2.1 Preparation and Assessment of bFGF‐Releasing Dura Mater

#### 2.1.1 Preparation of bFGF‐Releasing Dura Mater

The raw material used in the present study was a clinically applied bioabsorbable artificial dura mater (Durawave; Gunze, Osaka, Japan) composed of PGA (Yamaguchi et al. 2019). From this material, one‐layered and four‐layered dura maters (both of which measure 5 mm × 5 mm) were prepared. First, the pristine dura mater was cut into 5‐mm squares to prepare one‐layered dura mater. Second, the pristine dura mater was cut into 10‐mm squares, which were then folded into quarters. The resulting four‐layered dura mater was heated to 240°C at the four corners and central part with a soldering iron (FX‐888D; HAKKO Corporation, Osaka, Japan) for fusion welding.

The dura maters were exposed to oxygen plasma for 30 s at a power density of 0.5 W/cm^2^ in a 30 Pa O_2_ atmosphere using a compact ion etcher (FA‐1; Samco Inc., Kyoto, Japan). The plasma‐treated dura maters were sterilized with ethylene oxide gas, and subsequently incubated for 24 h in 0.3 mL of adsorption solutions with bFGF concentrations of 0, 4, and 12 µg/mL under shaking at 250 rpm using a shaking incubator (DWMaxM BR‐104P; Taitec Corporation, Saitama, Japan) maintained at 25°C. Before use, the adsorption solutions were prepared by dissolving the bFGF source (Fiblast; Kaken Pharmaceutical Co. Ltd., Tokyo, Japan) in 5 mL of a physiological solution (Otsuka Pharmaceutical Factory Inc., Tokushima, Japan) and subsequent dilution with phosphate‐buffered saline (PBS) (D‐PBS; Fujifilm Wako Pure Chemical Corporation, Tokyo, Japan). After shaking incubation for 24 h, the dura mater was removed from the adsorption solution, washed thrice with ultrapure water, and frozen at −80°C, followed by lyophilization using a freeze drier (FDS‐1000; Tokyo Rikakikai Co. Ltd., Tokyo, Japan). Lyophilized dura mater was stored at −30°C until required for the experiments.

Lyophilized dura mater prepared from the one‐layered dura mater using the adsorption solutions with bFGF concentrations of 0 and 4 µg/mL are referred to as FGF‐C and FGF‐L, respectively. Lyophilized dura mater prepared from the four‐layered dura mater using the adsorption solution with bFGF concentration of 12 µg/mL is referred to as FGF‐H. FGF‐C and FGF‐L were prepared in the same manner as reported previously, whereas the number of layers in FGF‐H was modified from that in a previous study (two‐layered dura mater was used) (Ito et al. 2025).

#### 2.1.2 bFGF‐Releasing Assay

The bFGF‐releasing capability of bFGF‐treated dura mater (FGF‐L and FGF‐H) was assayed as in our previous study (Ito et al. 2025). Each dura mater was incubated at 37°C in 2 mL of an acellular culture medium (DMEM; Thermo Fisher Scientific, Waltham, MA, USA) supplemented with 10% fetal bovine serum (Thermo Fisher Scientific) using a 24‐well culture plate in a humidified atmosphere containing 5% CO_2_ for up to 15 days (Ito et al. 2025, 2022; Salmina et al. 2024). After incubation for 3 h, 1 day, 4 days, 8 days, and 15 days, 150‐µL aliquots were sampled from the medium, and the residual medium was replenished with the equivalent volume (150 µL) of fresh medium. The sampled medium was subjected to an enzyme‐linked immunosorbent assay (ELISA) with a human FGF basic Quantikine ELISA kit (R&D Systems Inc., Minneapolis, MN, USA) to determine the bFGF concentration.

### 2.2 Animal Experiments

#### 2.2.1 Insertion of the Dura Mater on the Brain Injury

Eight‐week‐old male adult ICR mice (The Jackson Laboratory) were used. The animals were housed and handled in accordance with the guidelines established by the National Institute of Health Sciences of Japan. The animal experiments were conducted under the conditions approved by the Institutional Animal Ethics Committee (protocol code: 22‐016).

The mice were intraperitoneally anesthetized with ketamine (75 mg/kg) and xylazine (15 mg/kg). A midline scalp incision was made, and a 5‐mm‐diameter copper rod that had been pre‐cooled in liquid nitrogen was pressed onto the skull (2.5 mm posterior and 2.5 mm left‐lateral to the bregma as the center) for 30 s, inducing motor cortical lesions (Chiba et al. 2004; Murakami et al. 1999). A 5‐mm square section of the bone above the brain injury was removed using a drill. The bFGF‐treated dura mater (FGF‐L or FGF‐H) or the bFGF‐free dura mater (FGF‐C) as the control was inserted at the brain injury site, and the skin was sutured. The animals were randomly assigned to the FGF‐C, FGF‐L, and FGF‐H groups.

#### 2.2.2 Assessment of Neurological Functions

Neurological functions were assessed on Day 14 post‐brain injury (14 days after insertion of the dura mater) using cylinder and grid‐walking tests (Balkaya et al. 2013; Ito et al. 2025; Schaar et al. 2010). In the cylinder test, mice were placed in a 10‐cm‐diameter clear plastic cylinder and recorded for 5 min. In the grid‐walking test, mice were allowed to walk freely on a 20 × 20‐mm square wire grid, and their movements were recorded for 5 min. The right paw dragging ratio was defined as the percentage of right paw touches without slipping among the total number of paw touches. The number of foot faults was recorded when the contralesional paw slipped through an open grid, and the total number of contralateral paw steps was counted. The foot fault ratio was calculated as follows: (number of foot faults) / (total number of steps) × 100.

#### 2.2.3 Histological Assessment

The mice were euthanized by intraperitoneal administration of ketamine (75 µg/g) and xylazine (15 µg/g), followed by perfusion with PBS via the left ventricle. The extracted brain was cut into 2‐mm‐thick coronal slices, 4 mm from the frontal pole. The brain slices were fixed in 4% paraformaldehyde, embedded in paraffin, and sliced into 5‐µm‐thick coronal brain sections. The experiment was performed using FGF‐C (*n* = 13), FGF‐L (*n* = 13), and FGF‐H (*n* = 9). For chronological histological assessment, animals from each group were sacrificed on Day 7: FGF‐C, *n* = 5; FGF‐L, *n* = 5; and FGF‐H, *n* = 4. The remaining animals in each group were sacrificed on Day 14. One animal each from the FGF‐C and FGF‐L groups was excluded from histological assessment owing to tissue damage during brain resection on Day 14. The brain sections were stained with hematoxylin and eosin, and then subjected to microscopic scanning (Biozero, BZ8000; Keyence Corporation, Osaka, Japan). The brain injury area was quantified using ImageJ software (version 1.52a; National Institutes of Health, Bethesda, MD, USA) by subtracting the ipsilateral brain area from the contralateral brain area.

Fluorescent immunohistochemical staining was conducted using anti‐SRY‐box transcription factor 2 (SOX2), terminal transferase dUTP nick‐end labeling (TUNEL), occludin, and zonula occludens‐1 (ZO‐1) antibodies. The brain sections were treated with microwaves after deparaffinization, and then incubated at 4°C for 12 h with three primary antibodies: rabbit polyclonal anti‐mouse SOX2 (AB5603, 1:500 dilution, Merck Millipore, Burlington, MA, USA), rabbit polyclonal anti‐mouse occludin (13409‐1‐AP, 1:400 dilution, Proteintech, Wuhan, China), and rabbit anti‐mouse ZO‐1 (ab216880, 1:100 dilution, Abcam, Cambridge, UK). The sections were subsequently incubated with two secondary antibodies: goat anti‐rabbit IgG and Alexa Fluor 555 (A21429, 1:400, Thermo Fisher Scientific) at room temperature (approximately 25°C) for 60 min. The Cell Meter TUNEL apoptosis assay kit (cat #: 22844; AAT Bioquest, Sunnyvale, CA, USA) was used to detect apoptotic cells. The sections were counterstained with 4′,6‐diamidino‐2‐phenylindole (Roche Diagnostics, Mannheim, Germany). The stained sections were scanned using a fluorescence microscope (Biozero, BZ8000) and analyzed using ImageJ software. For quantitative analysis of SOX2‐positive cells, three different fields of view of the cortex around the brain injury were captured, and the mean number of SOX2‐positive cells per 1 mm^2^ was counted. For quantitative analysis of occludin‐positive, ZO‐1‐positive, and TUNEL‐positive cells, the percentage of positively stained (green fluorescence) cells over the total cells in each field of view of the cortex around the brain injury was calculated.

### 2.3 Statistical Analysis

All numerical data are presented as the mean ± standard error (SE). Statistical differences were analyzed using one‐way analysis of variance followed by the Dunnett post hoc test, which was used to detect significant differences between the control (FGF‐C) and bFGF‐treated (FGF‐L and FGF‐H) groups. SPSS (version 29.0; IBM Corp, Armonk, NY) was used to perform all analyses, and differences with *p* < 0.05 were considered statistically significant.

## 3 Results

**FIGURE 1:**
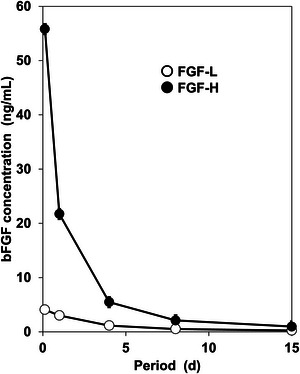
Basic fibroblast growth factor (bFGF) concentration changes in the medium during incubation of FGF‐L and FGF‐H for up to 15 days (*n* = 3, mean ± standard error).

The bFGF‐releasing assay confirmed that FGF‐L and FGF‐H have lower and higher bFGF‐releasing capabilities, respectively, corresponding to our previous result (Ito et al. 2025). The concentration of bFGF released from FGF‐H reached 55.81 ± 7.03 ng/mL at 3 h, which was approximately 14 times higher than that of FGF‐L (4.09 ± 0.54 ng/mL). A similar trend was observed throughout the assay period; FGF‐H consistently resulted in higher bFGF concentrations in the medium than FGF‐L for up to 15 days (Figure 1).

**FIGURE 2:**
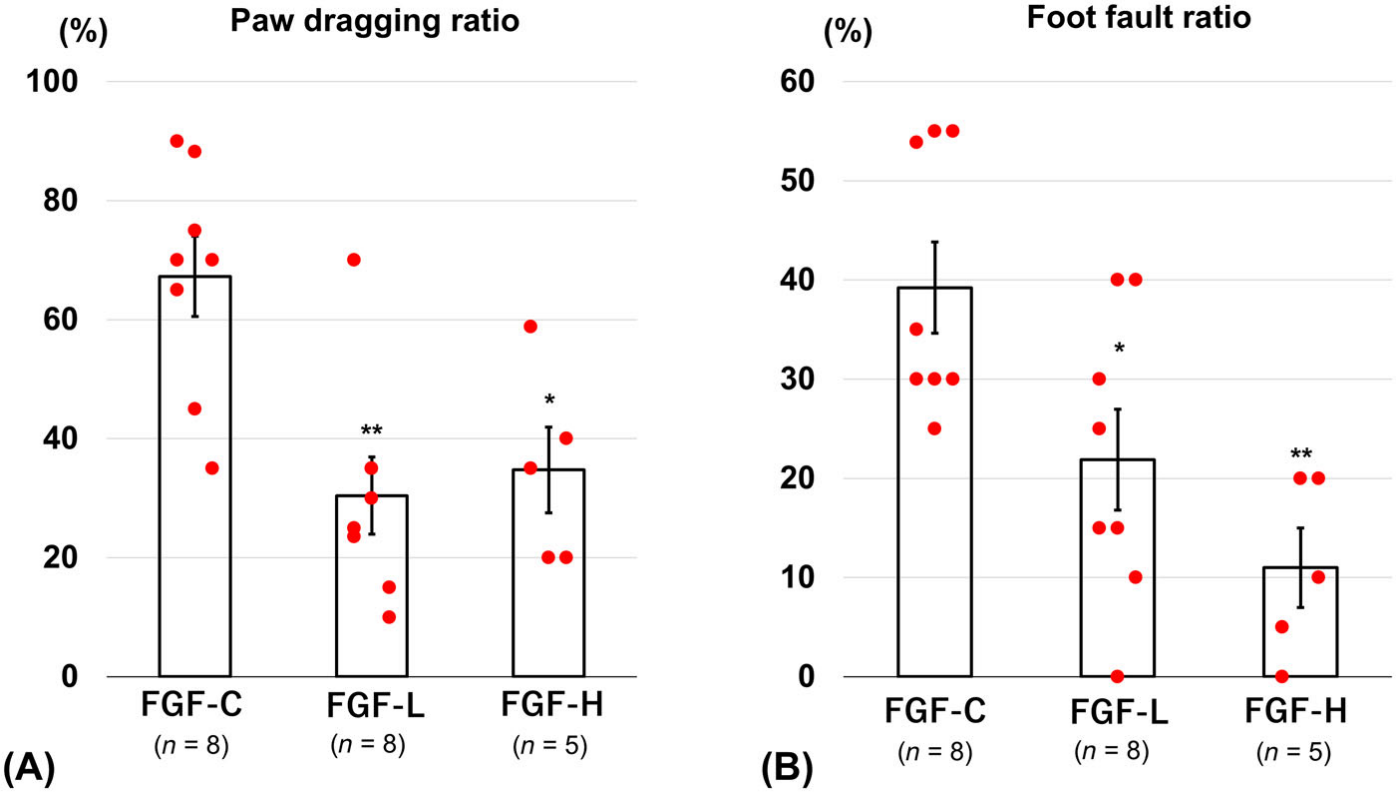
Neurological function assessment. (A) Right paw dragging ratio in the cylinder test and (B) foot fault ratio in the grid‐walking test on Day 14 after inserting the basic fibroblast growth factor (bFGF)‐releasing dura mater (lower bFGF‐release [FGF‐L] and higher bFGF‐release [FGF‐H]) and the bFGF‐free dura mater (FGF‐C) (mean ± standard error, ^*^ *p* < 0.05, and ^**^ *p* < 0.01).

Upper and lower extremity motor functions on Day 14 were significantly improved in the bFGF‐treated groups compared with those in the control group. The right paw dragging ratio in the cylinder test (measure of the upper extremity motor function) was significantly lower for FGF‐L (30.4 ± 6.5%, *p* = 0.001) and FGF‐H (34.8 ± 7.2%, *p* = 0.011) than that for FGF‐C (67.3 ± 6.8%) on Day 14 (Figure 2A). The foot fault ratio in the grid‐walking test (measure of the lower extremity motor function) was significantly lower for FGF‐L (21.9 ± 5.1%, *p* = 0.027) and FGF‐H (11.0 ± 4.0%, *p* = 0.002) than that for FGF‐C (39.2 ± 4.6%) on Day 14 (Figure 2B).

**FIGURE 3:**
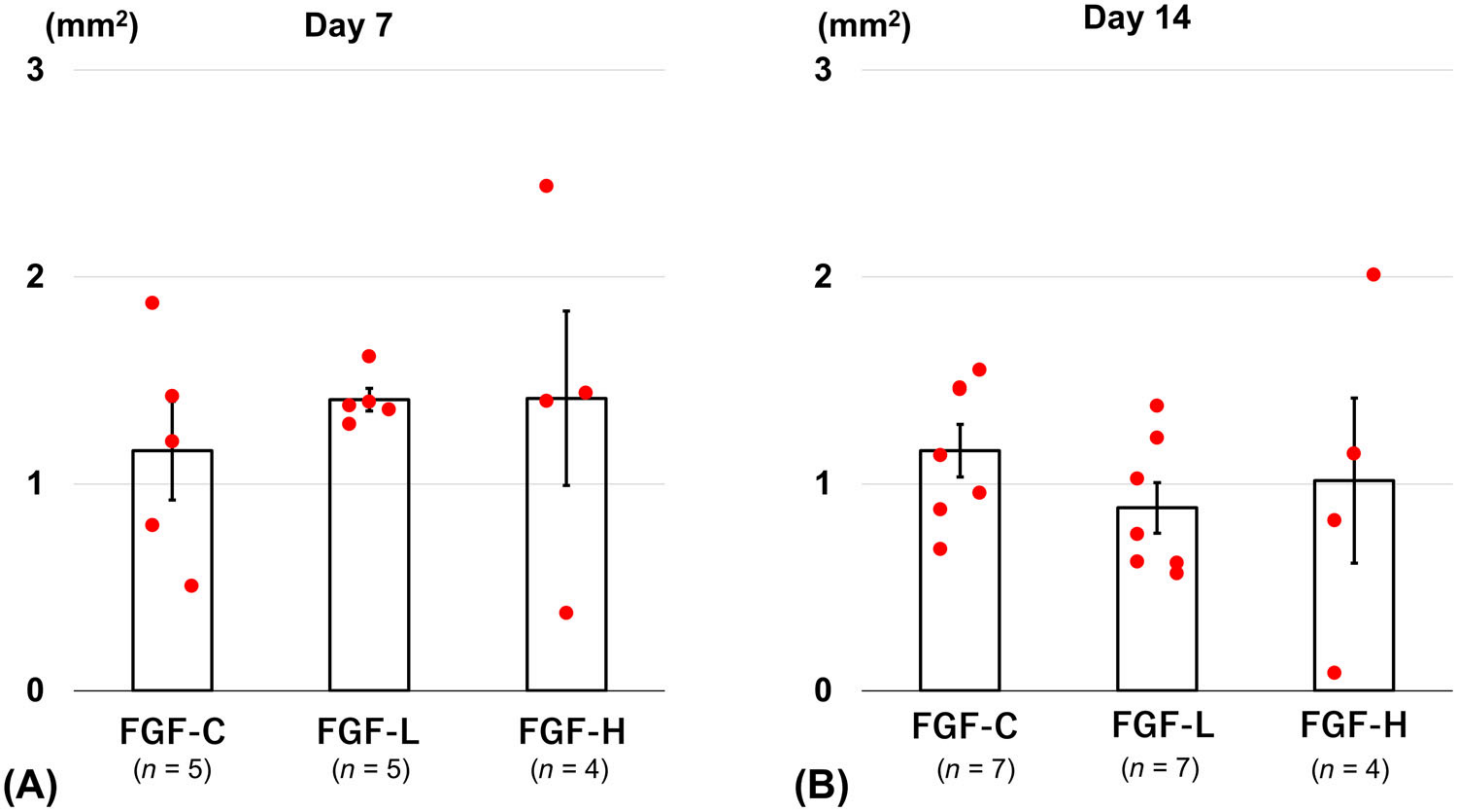
The brain injury area on Days (A) 7 and (B) 14 after inserting the basic fibroblast growth factor (bFGF)‐releasing dura mater (lower bFGF‐release [FGF‐L] and higher bFGF‐release [FGF‐H]) and the bFGF‐free dura mater (FGF‐C) (mean ± standard error).

No significant differences were found in the brain injury area on Day 7 or 14 between the bFGF‐treated and control groups. The brain injury areas on Day 7 were 1.16 ± 0.24, 1.41 ± 0.06, and 1.41 ± 0.42 mm^2^ in the FGF‐C, FGF‐L, and FGF‐H groups, respectively (Figure 3A). The brain injury areas on Day 14 were 1.16 ± 0.13, 0.88 ± 0.12, and 1.02 ± 0.40 mm^2^ in the FGF‐C, FGF‐L, and FGF‐H groups, respectively (Figure 3B).

**FIGURE 4:**
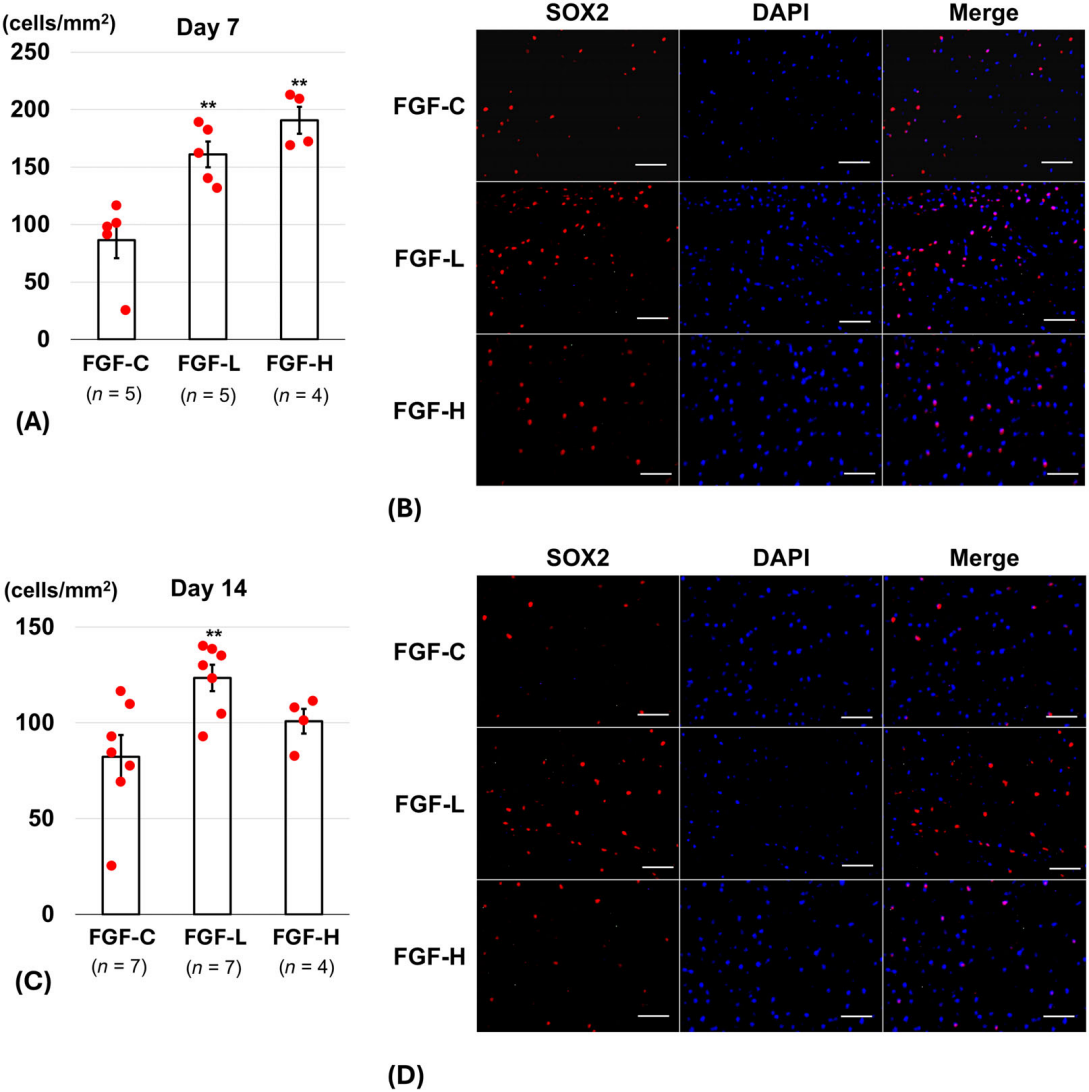
SRY‐box transcription factor 2 (SOX2)‐positive cells around the brain injury after inserting the basic fibroblast growth factor (bFGF)‐releasing dura mater (lower bFGF‐release [FGF‐L] and higher bFGF‐release [FGF‐H]) and the bFGF‐free dura mater (FGF‐C). (A,C) The number of SOX2‐positive cells in the cortex (per mm^2^) on Days (A) 7 and (C) 14 (mean ± standard error, ^**^ *p* < 0.01). (B,D) Representative fluorescence images (scale bar = 50 µm) of the cortex labeled with SOX2 (red) and 4′,6‐diamidino‐2‐phenylindole (DAPI) (blue) on Days (B) 7 and (D) 14, respectively.

The number of SOX2‐positive cells in the cortex on Day 7 was significantly larger in the FGF‐L (161.1 ± 11.3 cells/mm^2^, *p* = 0.003) and FGF‐H groups (190.8 ± 11.7 cells/mm^2^, *p* < 0.001) than in the FGF‐C group (86.4 ± 15.8 cells/mm^2^) (Figure 4A,B). On Day 14, however, only the FGF‐L group showed a significant increase in SOX2‐positive cells in the cortex (123.5 ± 6.8 cells/mm^2^, *p* = 0.008) compared with the FGF‐C group (82.2 ± 11.4 cells/mm^2^). The difference between the FGF‐H (100.9 ± 6.4 cells/mm^2^, *p* = 0.36) and FGF‐C groups was not significant (Figure 4C,D).

**FIGURE 5:**
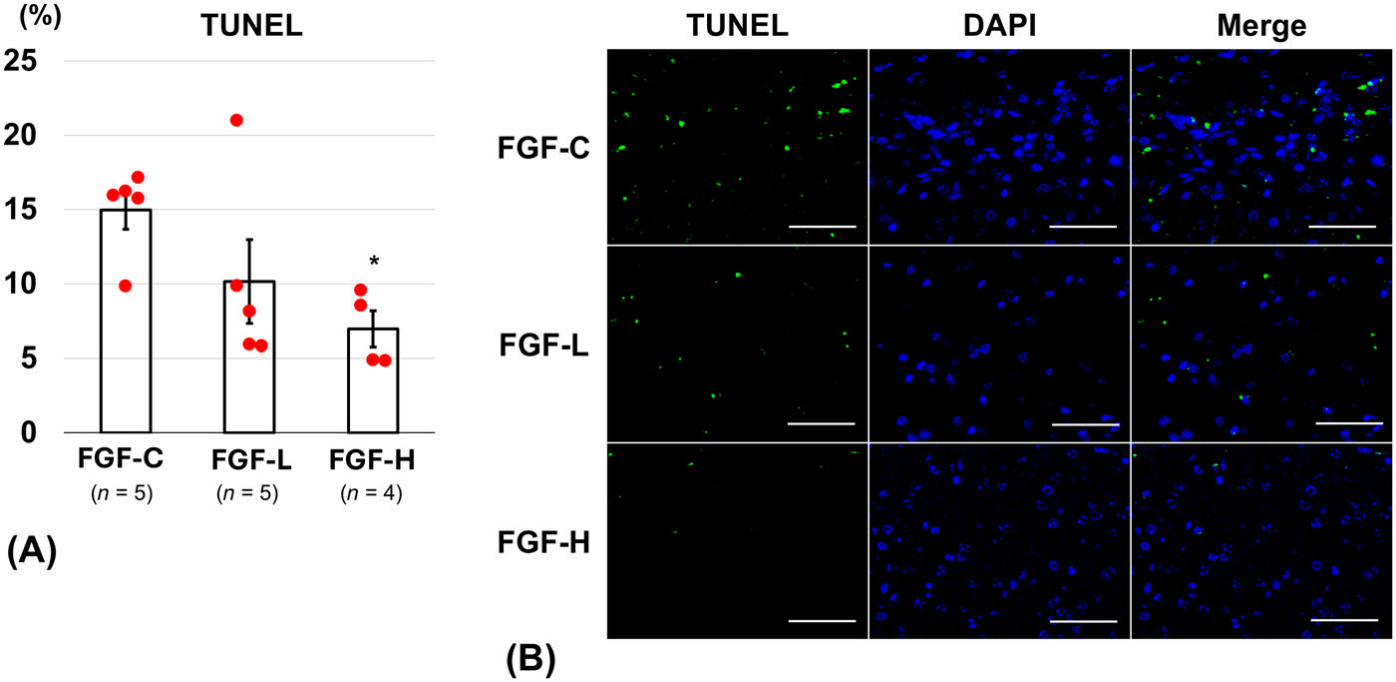
Terminal transferase dUTP nick‐end labeling (TUNEL)‐positive cells around the brain injury on Day 14 after inserting the basic fibroblast growth factor (bFGF)‐releasing dura mater (lower bFGF‐release [FGF‐L] and higher bFGF‐release [FGF‐H]) and the bFGF‐free dura mater (FGF‐C). (A) The ratio of TUNEL‐positive cells in the cortex (mean ± standard error). (B) Representative fluorescence images (scale bar = 50 µm) of the cortex labeled with TUNEL (green) and 4′,6‐diamidino‐2‐phenylindole (DAPI) (blue).

**FIGURE 6:**
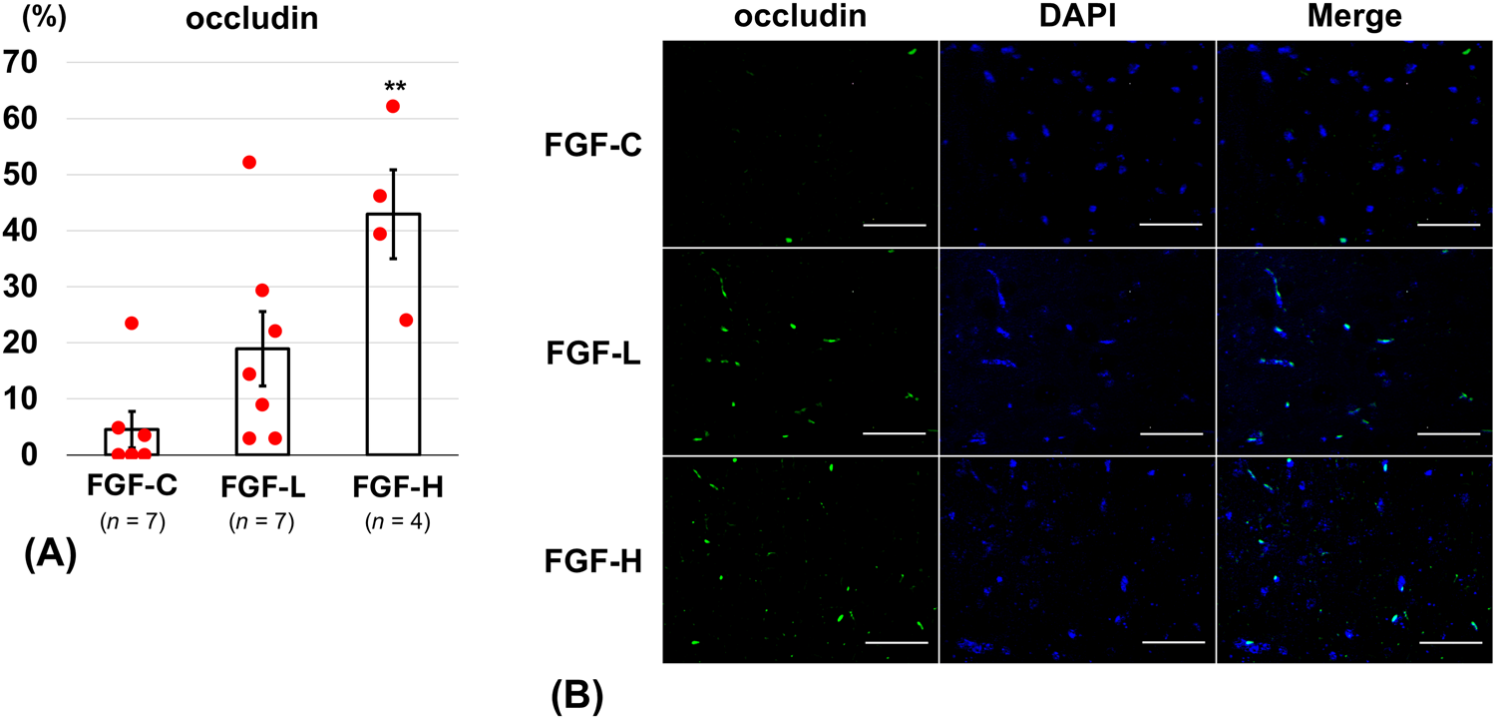
Occludin‐positive cells around the brain injury on Day 14 after inserting the basic fibroblast growth factor (bFGF)‐releasing dura mater (lower bFGF‐release [FGF‐L] and higher bFGF‐release [FGF‐H]) and the bFGF‐free dura mater (FGF‐C). (A) The ratio of occludin‐positive cells in the cortex (mean ± standard error). (B) Representative fluorescence images (scale bar = 50 µm) of the cortex labeled with occludin (green) and 4′,6‐diamidino‐2‐phenylindole (DAPI) (blue).

**FIGURE 7:**
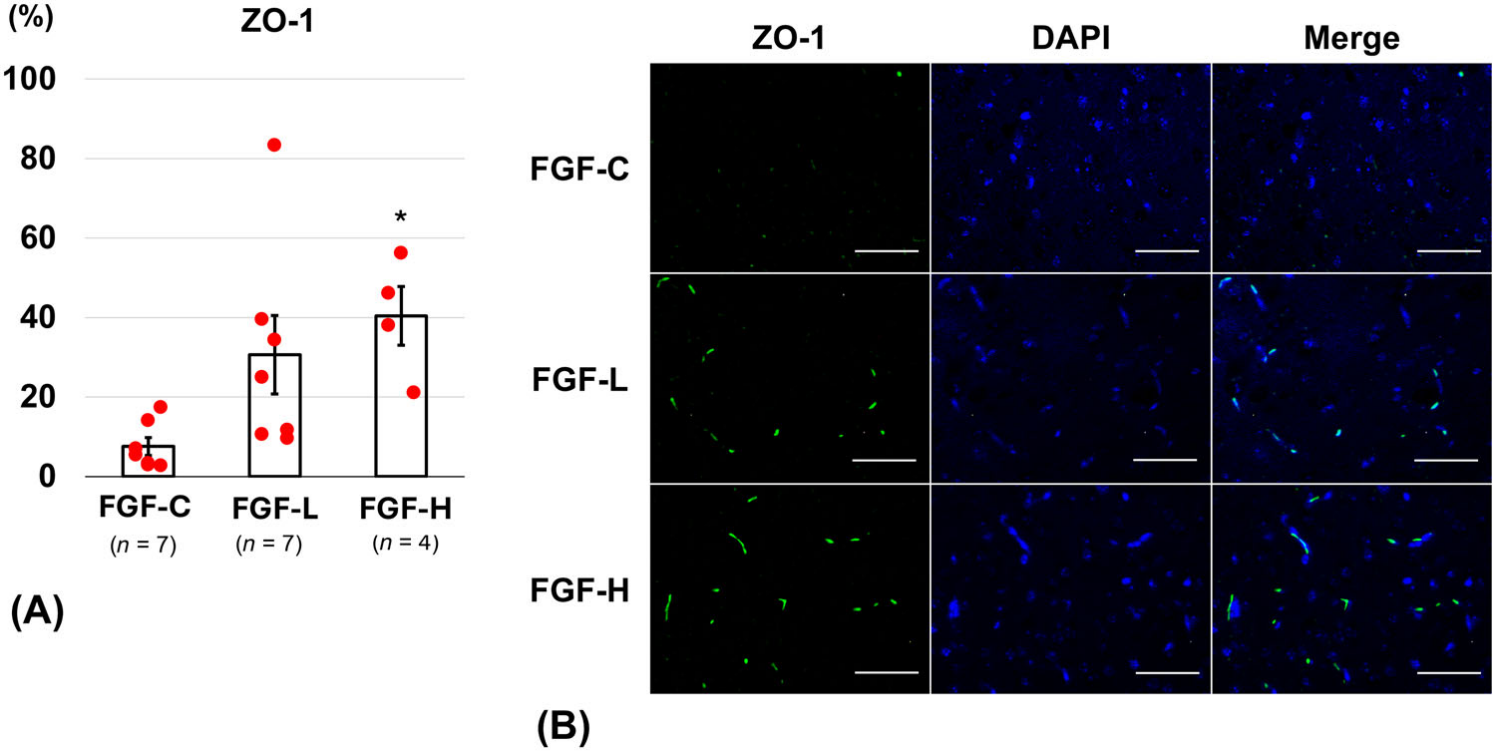
Zonula occludens‐1 (ZO‐1)‐positive cells around the brain injury on Day 14 after inserting the basic fibroblast growth factor (bFGF)‐releasing dura mater (lower bFGF‐release [FGF‐L] and higher bFGF‐release [FGF‐H]) and the bFGF‐free dura mater (FGF‐C). (A) The ratio of ZO‐1‐positive cells in the cortex (mean ± standard error). (B) Representative fluorescence images (scale bar = 50 µm) of the cortex labeled with ZO‐1 (green) and 4′,6‐diamidino‐2‐phenylindole (DAPI) (blue).

On Day 14, the FGF‐H group (7.0 ± 1.2%) revealed a significantly lower percentage of TUNEL‐positive cells than the FGF‐C group (15.0 ± 1.3%, *p* = 0.04) (Figure 5A,B). The FGF‐H group (42.9 ± 7.9%) showed a significantly higher percentage of occludin‐positive cells on Day 14 than the FGF‐C group (4.5 ± 3.2%, *p* = 0.001) (Figure 6A,B). The percentage of ZO‐1‐positive cells on Day 14 was significantly higher in the FGF‐H group (40.4 ± 7.4%) than in the FGF‐C group (7.6 ± 2.2%, *p* = 0.02) (Figure 7A,B). The percentages of TUNEL‐positive (10.1 ± 2.8%, *p* = 0.19), occludin‐positive (18.9 ± 6.6%, *p* = 0.14), and ZO‐1‐positive (30.6 ± 9.9%, *p* = 0.06) cells in the FGF‐L group were comparable to those in the FGF‐C group.

## 4 Discussion

This study confirmed that neurological functions significantly improved on Day 14 after brain injury in the bFGF‐treated groups compared with those in the control group. Histologically, the bFGF‐treated groups showed significantly more neural progenitor cells (SOX2‐positive cells) than the control group on Day 7. On Day 14, the FGF‐H group exhibited a substantial decrease in TUNEL‐positive cells and a significant increase in occludin‐positive and ZO‐1‐positive cells in comparison with the control group. However, no significant differences were observed in the cerebral contusion area between the control and bFGF‐treated groups on Day 14.

Functional disability after brain injury was observed in approximately half of all patients, highlighting the need for better treatments to fulfill unmet medical needs (Schneider et al. 2021). The main therapeutic target is controlling secondary brain injury. Several clinical studies have been conducted; however, the results are insufficient, and none have been translated into clinical practice (Thapa et al. 2021). Growth factors have been the focus of extensive research as potential therapeutic agents for stroke and brain injury because of their neuroprotective effects against glutamate excitotoxicity, oxidative damage, hypoxemia, and ischemia (Atkinson and Dickman 2023). However, most growth factors have short half‐lives in vivo, necessitating delivery systems to overcome this limitation. Animal‐derived and/or unapproved materials are often used as delivery carriers or stabilizers for growth factors; however, they pose clinical implementation challenges. In the present study, we used clinically approved artificial dura mater and bFGF to create the bFGF‐releasing dura mater via a simple two‐step process: surface plasma treatment and bFGF adsorption. The resulting bFGF‐releasing dura mater contained neither animal‐derived materials nor any other substances of safety concern. A similarly prepared dura mater previously released bFGF for at least 7 days and improved neurological function in a cerebral infarction model (Ito et al. 2025, 2022). The potential mechanisms underlying neural repair following ischemic stroke may include neuroprotective, neuroregenerative, angiogenic, and anti‐inflammatory effects of bFGF released from the dura mater. bFGF exerts neuroprotective effects by regulating apoptosis‐related proteins and suppressing oxidative stress via activation of the phosphoinositide 3‐kinase/protein kinase B pathway (Zhang et al. 2024). bFGF also stimulates angiogenesis and activates intrinsic neural progenitor cells, promoting synaptic connection formation (Duan et al. 2024). Furthermore, bFGF inhibits inflammatory cytokine secretion, suppresses microglial activation, and stabilizes BBB by inhibiting Ras homolog gene family member A (Huang et al. 2012; Tang et al. 2018). The present study demonstrated the therapeutic efficacy of the bFGF‐releasing dura mater for brain injuries.

In the present study, the bFGF‐releasing dura mater demonstrated a significant increase in neural progenitor cells (SOX2‐positive cells) in the cortex compared with the bFGF‐free dura mater on Day 7 after brain injury; however, the efficacy was attenuated (in FGF‐H) on Day 14. Neural progenitor cell proliferation typically peaks 7–14 days post‐brain injury (Urrea et al. 2007) and maximizes on Day 7 after bFGF administration (Ochi et al. 2016; Wada et al. 2003). Thus, the observed effect of bFGF‐releasing dura mater is consistent with that reported in previous studies. The stimulated neural progenitor cell proliferation should contribute to the improved neurological function in the bFGF‐treated groups (Nakatomi et al. 2002). On Day 14, a significant increase in SOX2‐positive cells was observed in the FGF‐L group compared with the FGF‐C group, whereas no significant difference was found between the FGF‐H and FGF‐C groups. The biphasic dose‐dependent response to growth factors is a reported phenomenon (Kanodia et al. 2014). Receptor saturation or inhibition of cellular responses due to overstimulation might be involved in the impaired efficacy observed in the FGF‐H group. To clarify the exact mechanism, further investigation is needed in the future.

Apoptosis and BBB impairments, evaluated as measures of secondary brain injury, were suppressed by the bFGF‐releasing dura mater (FGF‐H only). TUNEL‐positive cells, which are indicative of apoptotic cells, exhibited a marked decrease in the FGF‐H group compared with the FGF‐C group. Moreover, the FGF‐H group revealed more cells expressing the gap junction proteins occludin and ZO‐1 than the FGF‐C group, indicating BBB breakdown suppression by the bFGF‐releasing dura mater. The observed histological differences between the FGF‐H and FGF‐C groups should be attributed to bFGF released from the dura mater, which subsequently penetrated the damaged area. Intrathecally administered bFGF has been observed to migrate into the subarachnoid and perivascular space via the lymphocyte pathway, demonstrating prolonged biological effects compared with intravenously administered bFGF (Xie et al. 2015; Yang et al. 2013). The lack of an inhibitory effect of FGF‐L on apoptosis and BBB impairments is likely because of insufficient concentration of the released bFGF.

Here, the bFGF‐releasing dura mater was implanted immediately after brain injury; however, despite its efficacy on neurological functions, it did not reduce the brain injury area. That is, the bFGF‐releasing dura mater is ineffective in suppressing primary brain injury. According to previous studies, intravenous administration of bFGF reduced cerebral infarction and TBI (Dietrich et al. 1996; Fisher et al. 1995), indicating its efficacy in suppressing primary brain damage. Yet, intracisternal administration of bFGF via the ventricles did not reduce cerebral infarction (Kawamata et al. 1996). In the present study, the bFGF‐releasing dura mater was placed at the site of brain injury; hence, mechanism of action of the released bFGF should be similar to that of intrathecally administered bFGF. As mentioned above, the biological effect of intrathecally administered bFGF is likely to sustain longer than intravenously administered bFGF (Xie et al. 2015; Yang et al. 2013). This may account for the therapeutic effect of the bFGF‐releasing dura mater on secondary brain injury, such as BBB impairment and apoptosis.

The bFGF‐releasing dura mater may also be applied to other brain diseases beyond trauma. It is easily usable in craniotomy for brain diseases, for example, ischemic and hemorrhagic stroke, without prolonging the operative time or increasing invasion. As revealed in this study, the bFGF‐releasing dura mater has effects on neural progenitor cell increase, apoptosis inhibition, and BBB protection, suggesting its potential as a combination product that can reduce secondary brain damage around the extraction cavity while supporting the dural repair after surgeries.

This study has some limitations. First, FGF‐H (four‐layered dura mater) was thicker than FGF‐C and FGF‐L (one‐layered dura mater, equivalent to clinically used Durawave). The thicker dura mater in FGF‐H may have compressed brain tissue, affecting neurological regeneration and functions. The effect of thickness of dura mater must be clarified, and craniotomy should be extended to minimize brain pressure drainage. Second, this study lacks a comparison group of free bFGF administration without dura mater. In the brain, various cytokines are present, and expression of endogenous growth factors increases after brain injury. These cytokines may be adsorbed on the oxygen plasma‐treated PGA surface, potentially leading to a certain effect. Thus, a comparison of groups with and without PGA dura mater is required to better understand the mechanism of action. Third, evaluations of neurological functions and histological assessment were performed at limited time points, that is, on Day 7 or 14 after brain injury, with a limited sample size. Shorter‐term and longer‐term assessments and larger sample sizes are needed to better understand the clinical potential of the bFGF‐releasing dura mater. Further evaluations using larger animal models are also required for clinical translation.

## 5 Conclusion

The bFGF‐releasing artificial dura mater, composed of a PGA nonwoven fabric, increased neural progenitor cells, inhibited apoptosis and BBB impairments, and improved neurological functions in a brain injury mouse model. Further studies are required to determine its clinical application.

## Author Contributions

**Yoshiro Ito**: conceptualization, data curation, formal analysis, funding acquisition, investigation, methodology, project administration, resources, software, visualization, writing – original draft, writing – review and editing. **Ayako Oyane**: conceptualization, data curation, investigation, methodology, project administration, supervision, validation, writing – original draft, writing – review and editing. **Yuji Matsumaru**: supervision, writing – review and editing. **Eiichi Ishikawa**: supervision, writing – review and editing.

## Ethics Statement

All animal experiments, including surgical procedures, were approved by the University of Tsukuba Animal Ethics Committee (protocol code: 22‐016). This study adhered to internationally accepted standards for animal research, following the 3Rs principle. The Animal Research: Reporting of In Vivo Experiments guidelines were employed to report experiments involving live animals and promote ethical research practices. All animals were housed and handled according to the guidelines of the National Institute of Health Sciences of Japan. All animal procedures were performed in accordance with the US National Research Council “Guide for the Care and Use of Laboratory Animals.”

## Consent

The authors have nothing to report.

## Conflicts of Interest

The authors declare no conflicts of interest.

## Peer Review

The peer review history for this article is available at https://publons.com/publon/10.1002/brb3.71008.

## Data Availability

The datasets generated and analyzed during the current study are available from the corresponding author upon request.
